# Non-Doped Deep-Blue OLEDs Based on Carbazole-π-Imidazole Derivatives

**DOI:** 10.3390/ma14092349

**Published:** 2021-04-30

**Authors:** Pengfei Yu, Yin Xiao

**Affiliations:** School of Chemical Engineering and Technology, Tianjin Engineering Research Center of Functional Fine Chemicals, Tianjin University, Tianjin 300072, China; ypf19960057@163.com

**Keywords:** OLED, deep-blue emission, carbazole, imidazole

## Abstract

In this work, we designed and synthesized four bipolar blue-emitting materials with carbazole, imidazole, and biphenyl as donor, acceptor, and p bridge, respectively. The twisted phenylimidazole acceptor leads to a wider band-gap and hence deeper blue emission than the conjugated phenanthrimidazole acceptor. For the substituents on the carbazole donor, the t-butyl group could prevent the intramolecular charge transfer (ICT) process more effectively than the methoxy group. A non-doped deep-blue organic light-emitting diodes (OLED) is obtained with CIE coordinates of (0.159, 0.080), a maximum luminance of 11,364 cd/m^2^, and a maximum EQE of 4.43%.

## 1. Introduction

Organic light-emitting diodes (OLEDs) have great potential in the field of full-color flat-panel display and solid-state lighting due to their flexibility, low power consumption, and low cost [1,2]. Blue-emitting materials have significantly lagged behind their green and red counterparts, which still suffer from lower efficiency and color impurity [3,4,5,6]. Therefore, obtaining highly efficient fluorescent emitters in the blue spectrum range, especially with deep-blue emission, hold the key to the development of a full-color OLED display. Constructing bipolar molecules with donor-p-acceptor structures has been demonstrated as an effective method to afford highly efficient deep-blue emitting materials [7,8,9]. The electro-withdraw group facilitates obtaining a wide band-gap (Eg), resulting in deep-blue emitting. The donor could compensate the large hole-injection barriers at the hole-transporter/emitter junction and hence balance the carrier injection in devices [10,11,12,13,14]. Additionally, a suitable p-conjugation bridge could weaken the undesired intramolecular charge transfer (ICT) between the donor and accepter and hence avoid the bathochromic shift of the emission [15].

Herein, four carbazole-π-imidazole derivatives, 2-(4′-(3,6-di-tert-butyl-9H-carbazol-9-yl)-[1,1′-biphenyl]-4-yl)-1-phenyl-1H-phenanthro [9,10-d]imidazole (BCzB-PPI), 2-(4′-(3,6-dimethoxy-9H-carbazol-9-yl)-[1,1′-biphenyl]-4-yl)-1-phenyl-1H-phenanthro[9,10-d]imidazole (MoCzB-PPI), 3,6-di-tert-butyl-9-(4′-(1,4,5-triphenyl-1H-imidazol-2-yl)-[1,1′-biphenyl]-4-yl)-9H-carbazole(BCzB-PIM), and 3,6-dimethoxy-9-(4′-(1,4,5-triphenyl-1H-imidazol-2-yl)-[1,1′-biphenyl]-4-yl)-9H-carbazole (MoCzB-PIM) were synthesized. The carbazole is employed as the electron donor, which favors deep-blue emission due to its high singlet energy and benefits hole/electron transporting balance thanks to its efficient hole mobility. The phenanthroimidazole with a rigid planar structure is used as the electron acceptor to construct BCzB-PPI and MoCzB-PPI, which permit improvement of the thermal stability of the emitter. For comparison, a more twisted 4,5-diphenylimidazole is used instead of phenanthroimidazole to give BCzB-PIM and MoCzB-PIM. The biphenyl bridge with a twisted structure could be expected to reduce the conjugation of the whole molecule, which is in favor of blocking the ICT process. An OLED with BCzB-PPI as emitter affords deep-blue electroluminescence with a CIE coordinate of (0.157, 0.080) and maximum external quantum efficiency (EQE) of 4.43%.

## 2. Results and Discussions

**Scheme 1:**
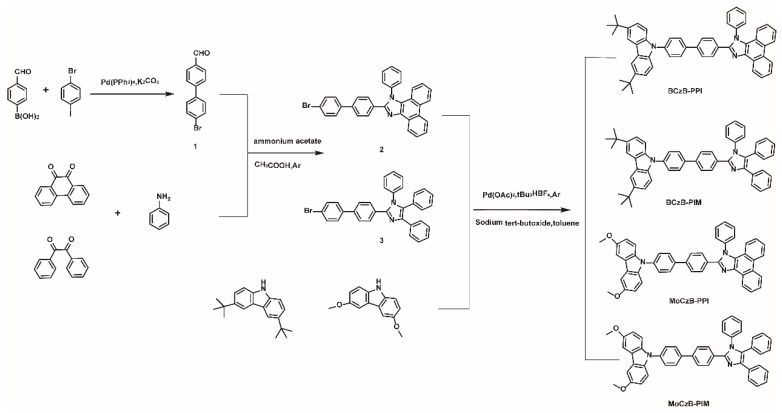
Molecular structure and synthetic routes of four compounds.

**Figure 1:**
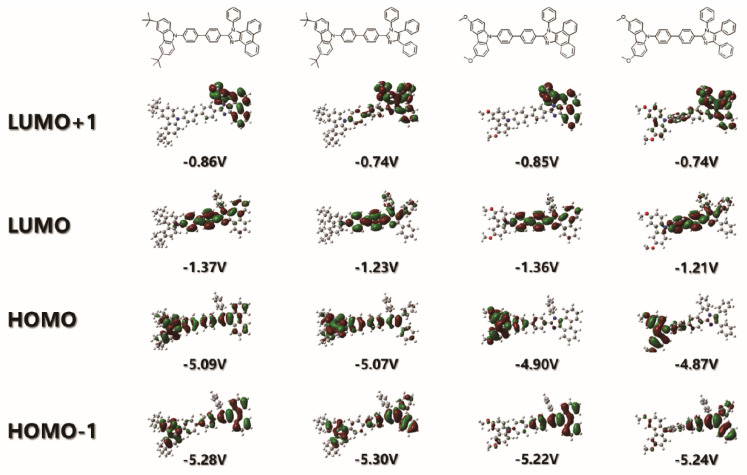
Relevant molecular orbital amplitude plots and energy levels of four compounds.

The molecular structure and synthetic routes are shown in Scheme 1, and detailed synthesis procedure and characterization are shown in Supporting Information. Density functional theory (DFT) calculation was carried out employing Gaussian 03 at the B3LYP/6–31G(d) level to obtain the electron distribution of the HOMO and LUMO energy levels of these compounds. As shown in Figure 1, the LUMO of all molecules is mainly located on the acceptor unit and the bridge. The HOMO of BCzB-PPI and BCzB-PIM with t-butyl moiety on the carbazole unit are almost localized on the whole molecule. While for MoCzB-PPI and MoCzB-PIM with methoxy group, the HOMO is mainly located on the electron-rich carbazole unit. The suitable HOMO-LUMO overlap of BCzB-PPI could be expected to prohibit the ICT process and afford a high fluorescence quantum yield. For t-butyl substituted compounds (BCzB-PPI and BCzB-PIM), it is allowed to transition from HOMO-1 to LUMO, from HOMO to LUMO + 1, and from HOMO to LUMO because of their effective overlaps. However, the transitions from the HOMO to the LUMO + 1 in methoxy substituted compounds (MoCzB-PPI and MoCzB-PIM) are nearly forbidden in the photo-absorption process, owing to their complete separation characters.

**Figure 2:**
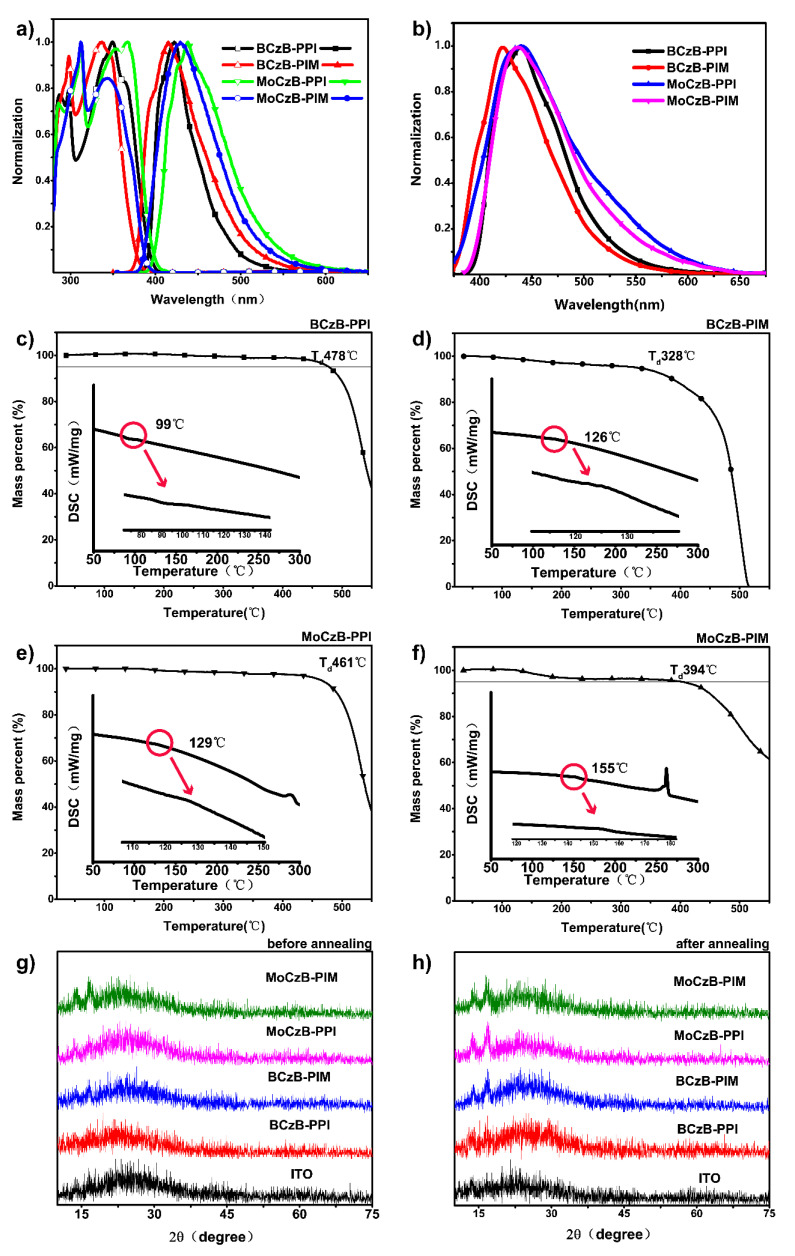
(**a**) Normalized absorption and emission spectra of four compounds in THF; (**b**) normalized emission of four compounds in the spin-coating film; (**c**–**f**) The DSC and TGA graphs of BCzB-PPI, BCzB-PIM, MoCzB-PPI, and MoCzB-PIM; (**h**,**g**) X-ray diffraction spectra of ITO, four compounds thin film on ITO substrate.

The UV-visible spectra and photoluminescence spectra of BCzB-PPI, BCzB-PIM, MoCzB-PPI, and MoCzB-PIM in THF solution and thin film are shown in Figure 2a,b, and related data are summarized in Table 1. The four compounds show similar UV-visible spectra with two absorption bands at 300–350 nm. The former one is attributed to n-π* transition of carbazole [16,17], while the latter one is attributed to the π-π* transition between the imidazole and the substituted benzene ring [9]. Compared to BCzB-PPI and MoCzB-PPI, the absorption peak around 330 nm of BCzB-PIM and MoCzB-PIM shows a significant blue shift, which is ascribed to the reduced conjugate range of BCzB-PIM and MoCzB-PIM caused by breaking one C–C bond of the phenanthrenere moiety. Moreover, BCzB-PPI and MoCzB-PPI have an absorption peak at the wavelength of 365 nm, which is assigned to the π-π* transition in the phenanthrimidazole group [9]. As shown in the photoluminescence spectra, the emission maximum of BCzB-PPI, MoCzB-PPI, BCzB-PIM, and MoCzB-PIM in THF is at 421, 438, 414, and 430 nm, respectively, corresponding to deep-blue emission. The blue shift of BCzB-PIM and MoCzB-PIM compared to BCzB-PPI and MoCzB-PPI arises from the small π-conjugated skeleton of non-coplanar phenylimidazole. A slight bathochromic shift (3–17 nm) can be observed in the film PL spectrum compared with THF solution, indicating the interaction of p-p stacking in the solid is effectively suppressed. The weaker red shift of PIM compounds than PPI compounds is ascribed to their more twisted structure. The PL spectra of these compounds in solvents with different polarities are shown in Figure S1 (Supporting Information). As expected, the BCzB-PPI and BCzB-PIM with t-butyl moiety on the carbazole unit exhibit slight bathochromic shifts as the solvent polarity increased, which suggests the weak ICT process benefited from their suitable HOMO-LUMO overlap as demonstrated by DFT calculation. The relatively large red shift of MoCzB-PPI and MoCzB-PIM with methoxy group in polar solvents is due to their relatively separated HOMO and LUMO. The fluorescence quantum yields of BCzB-PPI, MoCzB-PPI, BCzB-PIM, and MoCzB-PIM in THF were determined to be 96.22%, 92.24%, 77.45%, and 88.80%, respectively. All the compounds show a second-order exponential PL decay, as shown in Figure S2 (Supporting Information). The PL lifetimes of these compare from 1.37 to 2.99 ns. As we all know, the PL decay of fast fluorescence is nanoseconds, and the delayed fluorescence decays to hundreds of microseconds. It is proved that the four blue light materials are only ordinary fluorescence emission. Additionally, the optical band-gaps (Eg) of BCzB-PPI, BCzB-PIM, MoCzB-PPI, and MoCzB-PIM were calculated to be 3.08, 3.12, 3.01, and 3.08 V from the onset of UV-Vis absorption, respectively. Combined with cyclic voltammetry (CV) measurement (Figure S3, (Supporting Information)), the experimental HOMO and values of these compounds were also acquired as listed in Table 1. The wide Eg ensures deep-blue emission, and the low LUMO benefits the electron injection in the OLED device.

**Figure 3:**
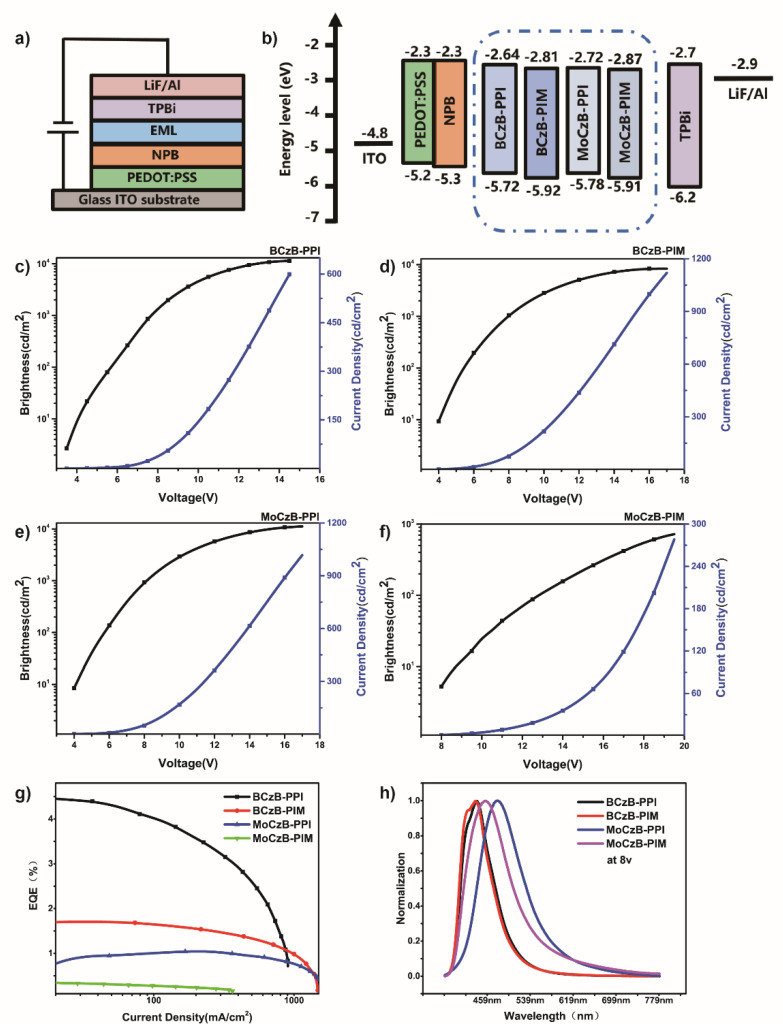
(**a**) The device structure; (**b**) energy level diagrams of the materials used in OLEDs; (**c**–**f**) current density-voltage-luminance curves of BCzB-PPI, BCzB-PIM, MoCzB-PPI, and MoCzB-PIM; (**g**) current density-EQE curves; (**h**) normalized electroluminance spectrum of the devices, inset EL spectra at 8 V.

In order to guarantee device application, the thermal property and the film morphology of these compounds were investigated. As shown in Figure 2c–f, the decomposition temperatures (T_d_, corresponding to 5 % weight loss) are determined to be as high as 478, 461, 328, and 394 °C for BCzB-PPI, MoCzB-PPI, BCzB-PIM, and MoCzB-PIM, respectively. The glass-transition temperatures (Tg) are measured to be 99, 129, 126, and 155 ℃. The better thermal stability of phenanthrimidazole-based compounds could be ascribed to the greater rigidity of the phenanthrimidazole group. The XRD spectra of the film before and after annealing are presented in Figure 2g,h. The films show the same XRD pattern before and after annealing at 90 °C for 1 h, suggesting their thermal stable amorphous structure [18]. More importantly, the BCzB-PPI exhibited the same featureless spectra as compared to the ITO substrate, which implies its weak crystallization on the ITO substrate hence guarantees high performance in the device [19]. Finally, a non-doped fluorescent OLED was fabricated with a structure of ITO/PEDOT/NPB/EML/TPBi/LiF/Al, in which PEDOT, NPB, and TPBi work as the anode buffer layer, hole transport layer, and electron transport layer, respectively. The energy level diagrams and device structure are displayed in Figure 3a,b. Key device parameters are summarized in Table 2. The devices present a low turn-on voltage around 3V except for OLED based on MoCzB-PIM. The higher turn-on voltage of the MoCzB-PIM device is attributed to the relatively large injection energy barrier between the light-emitting layer and the carrier injection layer. The devices with BCzB-PPI, BCzB-PIM, MoCzB-PPI, and MoCzB-PIM exhibit exhibited deep-blue EL spectra at 439, 437, 477, and 457 nm, respectively. As expected, the PIM compound exhibits deeper blue emissions than PPI compounds due to their small π-conjugated skeleton of twisted phenylimidazole. Importantly, the EL emission peak of the device based on BCzB-PPI shows only 1 nm redshift compared with its PL in films, which suggests the intermolecular interactions and the electrical field polarization in the excited states are effectively pressed. The BCzB-PPI-based non-doped device achieves the best performance with a maximum luminance of 11,364 cd/m^2^, a maximum EQE of 4.43%, and a Commission Internationale de l’Eclairage (CIE) coordinate of (0.157, 0.080) as deep-blue emission. The suitable performance of BCzB-PPI could be ascribed to its high PLQY, suitable thermal stability, and proper energy level. Compared with MoCzB-PPI and MoCzB-PIM, the suitable HOMO-LUMO overlap of BCzB-PPI and BCzB-PIM could prohibit the ICT process hence afford high PLQYs and lead to improvement of device performance. The higher HOMO level of BCzB-PPI than BCzB-PIM results in more efficient holes injection holes in the device.

Finally, the lifetime of the OLED with BCzB-PPI as the light-emitting layer was tested in the glovebox under N_2_. We tested the decay of the brightness over time when the initial brightness of the devices was 100, 500, and 1000 cd/m^2^, respectively (Figure S5). The half-lifetime (T50) was determined as 2.70, 0.242, and 0.086 h, respectively.

## 3. Conclusions

In conclusion, four carbazole-π-imidazole derivatives with blue emission were successfully synthesized, in which carbazole, imidazole, and biphenyl work as donor, acceptor, and p bridge, respectively. The BCzB-PIM and MoCzB-PIM with the non-conjugated phenylimidazole acceptor lead to deeper blue emission than BCzB-PPI and MoCzB-PPI with conjugated phenanthrimidazole acceptor. Compared with the methoxy group, the t-butyl moiety on the carbazole unit could prohibit the ICT process more effectively and lead to higher PLQY due to its suitable HOMO-LIMO overlap. Non-doped OLEDs with these compounds show blue emission. The device based on BCzB-PPI presents deep-blue emission with CIE coordinates of (0.159, 0.080), a maximum luminance of 11,364 cd/m^2,^ and a maximum EQE of 4.43%.

## Figures and Tables

**Table 1:** Basic properties of four compounds.

Compound	T_d_/T_g_ (°C)	$\lambda_{abs}^{a}$ (nm)	$\lambda_{em}^{a}$ (nm)	$\lambda_{em}^{b}$ (nm)	$\phi_{f}\;(\%)$	HOMO ^c^ (eV)	LUMO ^c^ (eV)	E_g_^d^ (eV)	*τ*^e^ (ns)
BCzB-PPI	478/99	344	421	438	96.22	−5.72	−2.64	3.09	1.43
BCzB-PIM	328/126	332,296	414	422	77.45	−5.92	−2.81	3.12	1.37
MoCzB-PPI	461/129	364,308	438	441	92.24	−5.78	−2.72	3.01	2.60
MoCzB-PIM	394/155	338,308	430	439	88.80	−5.91	−2.87	3.09	2.99

^a^ Absorption and PL: measured in 10^−5^ M THF. ^b^ PL: measured in neat film. ^c^ HOMO/LUMO energy levels estimated from cyclic voltammetry measurement. ^d^ Estimated by the absorption on set values. ^e^ PL lifetime in THF.

**Table 2:** Non-doped OLED device performances based on four compounds.

Compound	V_on_^a^ (V)	CE_max_^b^ (cd/A)	L_max_^c^ (cd/m^2^)	EQE ^d^ (%)	λ_EL_^e^ (nm)	CIE ^f^ (x,y)
BCzB-PPI	3.05	3.77	11,364	4.43	439	(0.157, 0.080)
BCzB-PIM	3.11	1.70	8342	1.72	437	(0.159, 0.075)
MoCzB-PPI	3.12	1.93	11,198	1.05	477	(0.255, 0.244)
MoCzB-PPI	3.12	1.93	11,198	1.05	477	(0.255, 0.244)
MoCzB-PIM	7.14	0.50	722	0.34	457	(0.196, 0.216)

^a^ Turn-on voltage recorded at the luminance of 1 cdm2. ^b^ Maximum luminous efficiency. ^c^ Maximum luminance. ^d^ Maximum external quantum efficiency. ^e^ Maximum peak of EL spectra. ^f^ Measured at 8 V.

## Data Availability

The data presented in this study are available on request from the corresponding author.

## Supplementary Materials

The following are available online at https://www.mdpi.com/article/10.3390/ma14092349/s1. Figure S1. Normalized emission of BCzB-PPI (a), BCzB-PIM (b), MoCzB-PPI (c) and MoCzB-PIM (d) in different solutions: toluene, DCM, THF, and DMF; Figure S2. The decay behavior of BCzB-PPI (a), BCzB-PIM (b), MoCzB-PPI (c) and MoCzB-PIM (d) in degassed THF by using time-correlated single photon counting method; Figure S3. Cyclic voltammograms of four compounds, the potentials are calibrated against Fc/ Fc+ internal standard; Figure S4. Current density-current efficiency curves; Figure S5. The lifetime (T50) of BCzB-PPI OLED at initial luminance of 100 (a), 500 (b) and 1000 cd/m^2^ (c); Figure S6. 1H NMR (a) and 13C NMR (b) of BCzB-PPI; Figure S7. 1H NMR (a) and 13C NMR (b) of BCzB-PIM; Figure S8. 1H NMR (a) and 13C NMR (b) of MoCzB-PPI; Figure S9. 1H NMR (a) and 13C NMR (b) of MoCzB-PIM.

## References

[1] Yang X., Xu X., Zhou G. (2015). Recent advances of the emitters for high performance deep-blue organic light-emitting diodes. J. Mater. Chem. C.

[2] Mallesham G., Swetha C., Niveditha S., Mohanty M.E., Babu N.J., Kumar A., Bhanuprakash K., Rao V.J. (2015). Phosphine oxide functionalized pyrenes as efficient blue light emitting multifunctional materials for organic light emitting diodes. J. Mater. Chem. C.

[3] D’Andrade B.W., Forrest S.R. (2004). White organic light-emitting devices for solid-state lighting. Adv. Mater..

[4] Chou H.H., Cheng C.H. (2010). A Highly Efficient Universal Bipolar Host for Blue, Green, and Red Phosphorescent OLEDs. Adv. Mater..

[5] Chen C.H., Hsu L.C., Rajamalli P., Chang Y.W., Wu F.I., Liao C.Y., Chiu M.J., Chou P.Y., Huang M.J., Chu L.K. (2014). Highly efficient orange and deep-red organic light emitting diodes with long operational lifetimes using carbazole-quinoline based bipolar host materials. J. Mater. Chem. C.

[6] Fan C.H., Sun P.P., Su T.H., Cheng C.H. (2011). Host and Dopant Materials for Idealized Deep-Red Organic Electrophosphorescence Devices. Adv. Mater..

[7] Jia Y., Wu S., Zhang Y., Fan S., Zhao X., Liu H., Dong X., Wang S., Li X. (2019). Achieving non-doped deep-blue OLEDs by applying bipolar imidazole derivatives. Org. Electron..

[8] Jia Y., Zhang Y.T., Fan S.G., Wu S., Zhan X.M., Wang S.R., Li X.G. (2019). A novel bipolar carbazole/ phenanthroimidazole derivative for high efficiency nondoped deep-blue organic light-emitting diodes. Org. Electron..

[9] Gao Z., Wang Z., Shan T., Liu Y., Shen F., Pan Y., Zhang H., He X., Lu P., Yang B. (2014). High-efficiency deep blue fluorescent emitters based on phenanthro 9,10-d imidazole substituted carbazole and their applications in organic light emitting diodes. Org. Electron..

[10] Yuan Y., Chen J.-X., Lu F., Tong Q.-X., Yang Q.-D., Mo H.-W., Ng T.-W., Wong F.-L., Guo Z.-Q., Ye J. (2013). Bipolar Phenanthroimidazole Derivatives Containing Bulky Polyaromatic Hydrocarbons for Nondoped Blue Electroluminescence Devices with High Efficiency and Low Efficiency Roll-Off. Chem. Mater..

[11] Chang C.-H., Kuo M.-C., Lin W.-C., Chen Y.-T., Wong K.-T., Chou S.-H., Mondal E., Kwong R.C., Xia S., Nakagawa T. (2012). A dicarbazole-triazine hybrid bipolar host material for highly efficient green phosphorescent OLEDs. J. Mater. Chem..

[12] Benor A., Takizawa S.Y., Perez-Bolivar C., Anzenbacher P. (2010). Energy barrier, charge carrier balance, and performance improvement in organic light-emitting diodes. Appl. Phys. Lett..

[13] Lee S., Kim K.H., Limbach D., Park Y.S., Kim J.J. (2013). Low Roll-Off and High Efficiency Orange Organic Light Emitting Diodes with Controlled Co-Doping of Green and Red Phosphorescent Dopants in an Exciplex Forming Co-Host. Adv. Funct. Mater..

[14] Huang J.H., Su J.H., Li X., Lam M.K., Fung K.M., Fan H.H., Cheah K.W., Chen C.H., Tian H. (2011). Bipolar anthracene derivatives containing hole- and electron-transporting moieties for highly efficient blue electroluminescence devices. J. Mater. Chem..

[15] Chen X., Zhao J.W., Zheng X.H., Zhu J.J., Yang G.X., Tang S.S., Tong Q.X., Tao S.L. (2019). Efficient deep blue OLEDs with extremely low efficiency roll-off at high brightness based on phenanthroimidazole derivatives. Chin. Chem. Lett..

[16] Zhao L., Wang S., Shao S., Ding J., Wang L., Jing X., Wang F. (2015). Stable and efficient deep-blue terfluorenes functionalized with carbazole dendrons for solution-processed organic light-emitting diodes. J. Mater. Chem. C.

[17] Huang Y., Du X., Tao S., Yang X., Zheng C.-J., Zhang X., Lee C.-S. (2015). High efficiency non-doped deep-blue and fluorescent/phosphorescent white organic light-emitting diodes based on an anthracene derivative. Synth. Met..

[18] Fan S., You J., Miao Y., Wang H., Bai Q., Liu X., Li X., Wang S. (2016). A bipolar emitting material for high efficient non-doped fluorescent organic light-emitting diode approaching standard deep blue. Dye. Pigment..

[19] Zhang Y.T., Wang X., Li X.G., Wang S.R., Pan Y.C., Zhong Z.M., Ying L., Xiao Y. (2018). A thermally cross-linked hole-transporting film with the remarkable solvent resistance for solution-processed OLEDs. Org. Electron..

