# Supplementary material for: Non-Doped Deep-Blue OLEDs Based on Carbazole-π-Imidazole Derivatives
Source: Materials (Basel). 2021 Apr 30;14(9):2349. doi: 10.3390/ma14092349 (PMC8125583; doi:10.3390/ma14092349)
Supplement: Supplementary file 1 [file materials-14-02349-s001.zip › materials-1189967-supplementary.pdf]

materials

MDPI

Communication

Non-Doped Deep-Blue OLEDs Based on Carbazole-π-Imidazole Derivatives

Pengfei Yu and Yin Xiao *

School of Chemical Engineering and Technology, Tianjin Engineering Research Center of Functional Fine Chemicals, Tianjin University, Tianjin 300072, China; ypf19960057@163.com
* Correspondence: xiaoyin@tju.edu.cn

Abstract: In this work, we designed and synthesized four bipolar blue-emitting materials with carbazole, imidazole, and biphenyl as donor, acceptor, and p bridge, respectively. The twisted phenylimidazole acceptor leads to a wider band-gap and hence deeper blue emission than the conjugated phenanthrimidazole acceptor. For the substituents on the carbazole donor, the t-butyl group could prevent the intramolecular charge transfer (ICT) process more effectively than the methoxy group. A non-doped deep-blue organic light-emitting diodes (OLED) is obtained with CIE coordinates of (0.159, 0.080), a maximum luminance of 11364 cd/m², and a maximum EQE of 4.43%.

Keywords: OLED; deep-blue emission; carbazole; imidazole

Citation: Yu , P.; Xiao , Y. Non-Doped Deep-Blue OLEDs Based on Carbazole-p-Imidazole Derivatives. *Materials* **2021**, *14*, 2349. https://doi.org/10.3390/ma14092349

Academic Editor: Massimo Cocchi

Received: 2 April 2021
Accepted: 28 April 2021
Published: 30 April 2021

Publisher's Note: MDPI stays neutral with regard to jurisdictional claims in published maps and institutional affiliations.

Copyright: © 2021 by the authors. Licensee MDPI, Basel, Switzerland. This article is an open access article distributed under the terms and conditions of the Creative Commons Attribution (CC BY) license (http://creativecommons.org/licenses/by/4.0/).

1. Material

(4-formylphenyl）boronic acid, 1-bromo-4-iodobenzene, aniline, phenanthrene-9,10-dione, benzil, 3,6-di-tert-butyl-9H-carbazole and other reagents were purchased from Tianjin C&S Biochemical Technology Co., Ltd.. $Pd(OAc)_2$ and $Pd(PPh_3)_4$ were purchased from Aladdin. Toluene was distilled from sodium/benzophenone under argon (Ar) atmosphere.

2. Synthesis

4′-bromo-[1,1′-biphenyl]-4-carbaldehyde (1): Under the protection of nitrogen, (4-formylphenyl）boronic acid (2.25g, 15 mmol), 1-bromo-4-iodobenzene (4.67g, 16.5 mmol), $Pd(PPh_3)_4$ (173.4 mg, 1 mol%), K_2CO_3 aqueous solution (6.2 g, 100 ml H2O) , Toluene (60 mL), ethanol (30 mL) were added to a flask, and reacted at 90 °C for 12 h. After the reaction was completed, it was cooled to room temperature and extracted with dichloromethane. The organic phase was purified with column chromatograph to afford **1** as white solid (3.02 g). Yield：78%. ^{1}H NMR (400 MHz, CDCl3) δ 10.06 (s, 1H), 7.96 (d, 2H, J =8.00 Hz), 7.72 (d, 2H, J = 8.00 Hz), 7.61 (d, 2H, J = 8.00 Hz), 7.50 (d, 2H, J = 8.00 Hz) ppm.

2-(4′-bromo-[1,1′-biphenyl]-4-yl)-phenyl-1H-phenanthro[9,10-d]imidazole (2): 4′-bromo-[1,1′-biphenyl]-4-carbaldehyde (2.6 g, 10 mmol), phenanthrene-9,10-dione (2.1 g, 10 mmol), aniline (930 mg, 10 mmol), ammonium acetate (4.7 g, 60 mmol), acetic acid (120 mL) were added to a flask and refluxed at 120 °C for 12 h under N_2. After cooled to room temperature, reaction liquid was dropped into water to precipitated. The solids were washed with cold methanol for three times to afford **2** (4.8 g) as light yellow solids. Yield: 92%. ^{1}H NMR (400 MHz, CDCl3) δ 8.88 (d, 1H, J=8.00 Hz), 8.78 (d, 1H, J =8.00 Hz), 8.72 (d, 1H, J = 8.00 Hz), 7.76(t, 1H, J = 8.00 Hz), 7.69-7.59 (m, 6H), 7.58-7.40 (m, 9H), 7.25 (t, 1H, J = 8.00 Hz), 7.19 (d, 1H, J = 8.00 Hz) ppm

2-(4′-bromo-[1,1′-biphenyl]-4-yl)-1,4,5-triphenyl-1H-imidazole (3): The reaction step was the same as the synthesis of 2-(4′-bromo-[1,1′-biphenyl]-4-yl)-phenyl-1H-phenanthro[9,10-d]imidazole. Replace phenanthrene-9,10-dione with benzil to obtain 4.95g of

gray solid. Yield：95%. ^{1}H NMR (400 MHz, CDCl3) δ 7.62-7.60 (m, 6H), 7.55-7.49(m, 12H), 7.45-7.42 (m, 11H) ppm

2-(4′-(3,6-di-tert-butyl-9H-carbazol-9-yl)-[1,1′-biphenyl]-4-yl)-1-phenyl-1H-phenanthro[9,10-d]imidazole (BCzB-PPI) : The 2-(4′-bromo-[1,1′-biphenyl]-4-yl)-1,4,5-triphenyl-1H-imidazole (4.7 g, 9 mmol), 3,6-di-tert-butyl-9H-carbazole (2.5 g, 9 mmol), $Pd(OAc)_2$ (60 mg, 3 mol%), $tBu_3P·HBF_4$ (146 mg, 6 mol%)), sodium tert-butoxide (1.8 g, 18mmol), toluene (150 mL) were added to the flask and heated at 120°C to reflux for 14 h under N_2. Then, water was added. After extraction with dichloromethane, the organic phase was purified with column chromatograph to afford **BCzB-PPI** 5.20 g as white solid. Yield :80%. ^{1}HNMR (400 MHz, CDCl3): δ 8.91 (d, 1H, J = 8.00 Hz), 8.79(d, 1H, J =8.00 Hz), 8.73 (d, 1H, J =8.00 Hz), 8.15 (s, 2H), 7.80-7.39 (m, 20H), 7.21-7.30 (m, 2H), 1.47(s, 18H) ppm.^{13}CNMR（600M，$CDCl_3$）：δ 150.70(s), 143.22(s), 140.61(s), 139.37(s), 139.06(s), 138.88(s), 137.94(s), 130.50(s), 130.13(s), 130.09(s), 129.56(s), 129.41(s), 128.56(s), 128.54(s), 128.49(s), 127.58(s), 127.47(s), 127.31(s), 126.99(s), 126.56(s), 125.93(s), 125.19(s), 124.39(s), 123.91(s), 123.71(s), 123.40(s), 123.28(s), 123.06(s), 121.12(s), 116.53(s), 109.50(s), 35.00(s), 32.29(s). MALDI-TOF (*m*/*z*): [M^+] calcd for $C_{53}H_{45}N_3$: 723.96; Found: 724.23. Anal. Calcd(%) for $C_{53}H_{45}N_3$: C, 87.93; H, 6.27; N, 5.80. Found: C, 87.88; H, 6.31; N, 5.73.

Synthesis of BCzB-PIM（3，6-di-tert-butyl-9-(4′-(1,4,5-triphenyl-1H-imidazol-2-yl)-[1,1′-biphenyl]-4-yl)-9H-carbazole) : The reaction step was the same as the synthesis of BCzB-PPI. Replace 2-(4′-bromo-[1,1′-biphenyl]-4-yl)-phenyl-1H-phenanthro[9,10-d]imidazole with 2-(4′-bromo-[1,1′-biphenyl]-4-yl)-1,4,5-triphenyl-1H-imidazole to obtain 2.70g of gray solid. Yield :41.3%. ^{1}HNMR (400 MHz, CDCl3): δ 8.14 (d, 2H, J = 8.14 Hz), 7.78(d, 2H, J =8.00 Hz), 7.65-7.38 (m, 14H), 7.33-7.20 (m, 7H), 7.17-7.11 (m, 4H), 1.47(s, 18H)ppm. ^{13}CNMR（600M，$CDCl_3$）：δ 146.68(s), 143.21(s), 139.41(s), 139.05(s), 137.89(s), 131.42(s), 131.37(s), 129.74(s), 129.53(s), 129.05(s), 128.75(s), 128.69(s), 128.50(s), 127.79(s), 127.26(s), 127.17(s), 126.98(s), 123.91(s), 123.70(s), 116.53(s), 109.50(s), 35.02(s), 32.30(s). MALDI-TOF (*m*/*z*): [M^+] calcd for $C_{53}H_{47}N_3$: 725.98; Found: 726.16. Anal. Calcd(%) for $C_{53}H_{47}N_3$: C, 87.69; H, 6.53; N, 5.79. Found: C, 87.67; H, 6.59; N, 5.84.

Synthesis of MoCzB-PPI（2-(4′-(3,6-dimethoxy-9H-carbazol-9-yl)-[1,1′-biphenyl]-4-yl)-1-phenyl-1H-phenanthro[9,10-d]imidazole) : The reaction step was the same as the synthesis of BCzB-PPI. Replace 3,6-di-tert-butyl-9H-carbazole with 3,6-dimethoxy-9H-carbazole to obtain 1.30g of gray solid. Yield :21.5%. ^{1}HNMR (400 MHz, CDCl3): δ 8.93 (d, 1H, J = 8.00 Hz), 8.80(d, 1H, J =8.00 Hz), 8.73 (d, 1H, J =8.00 Hz), 7.80-7.52 (m, 16H), 7.39 (d, 1H, J = 8.00 Hz), 7.21-7.20(m, 5H), 7.07-7.04(m, 2H), 3.96(s, 6H) ppm. ^{13}CNMR（600M，$CDCl_3$）：δ 154.38(s), 138.92(s), 137.98(s), 136.42(s), 130.56(s), 130.23(s), 130.17(s), 129.63(s), 129.43(s), 128.59(s), 127.65(s), 127.10(s), 126.61(s), 126.02(s), 125.28(s), 124.42(s), 124.01(s), 123.41(s), 123.25(s), 123.13(s), 121.16(s), 115.50(s), 111.02(s), 56.39(s). MALDI-TOF (*m*/*z*): [M^+] calcd for $C_{47}H_{33}N_3O_2$: 671.80; Found: 672.10. Anal. Calcd(%) for $C_{47}H_{33}N_3O_2$: C, 84.03; H, 4.95; N, 6.25; O, 4.76. Found: C, 83.98; H, 5.01; N, 6.28; O, 4.79.

Synthesis of MoCzB-PIM（3,6-dimethoxy-9-(4′-(1,4,5-triphenyl-1H-imidazol-2-yl)-[1,1′-biphenyl]-4-yl)-9H-carbazole) : The reaction step was the same as the synthesis of MoCzB-PPI. Replace 2-(4′-bromo-[1,1′-biphenyl]-4-yl)-phenyl-1H-phenanthro[9,10-d]imidazole with 2-(4′-bromo-[1,1′-biphenyl]-4-yl)-1,4,5-triphenyl-1H-imidazole to obtain 1.9 g of gray solid. Yield :31.4%. ^{1}HNMR (400 MHz, CDCl3): δ 7.78 (d, 2H, J = 8.00 Hz), 7.65-7.38 (m, 10H), 7.38 (d, 2H, J = 8.00 Hz), 7.34-7.22 (m, 9H), 7.17-7.11 (m, 4H), 7.07-7.04 (m, 2H), 3.96(s, 6H) ppm. ^{13}CNMR（600M，$CDCl_3$）：δ 154.34(s), 146.72(s), 139.07(s), 137.79(s), 136.42(s), 131.39(s), 129.62(s), 129.46(s), 128.75(s), 128.66(s), 128.63(s), 128.51(s), 128.46(s), 128.29(s), 127.68(s), 127.05(s), 126.94(s), 126.92(s), 123.97(s), 115.47(s), 111.00(s), 56.37(s). MALDI-TOF (*m*/*z*): [M^+] calcd for $C_{47}H_{35}N_3O_2$: 673.82; Found: 674.16. Anal. Calcd(%) for $C_{47}H_{35}N_3O_2$: C, 83.78; H, 5.24; N, 6.24; O, 4.75. Found: C, 83.82; H, 5.26; N, 6.27; O, 4.71.

3. Characterization

^{1}H NMR measurements were conducted on a 400M liquid nuclear magnetic resonance spectrometer. Mass spectra (MS) data were obtained on a matrix-assisted laser desorption-tandem time-of-flight mass spectrometry. Absorption spectra and Photoluminescence spectra were recorded on a Thermo Evolution 300 UV-Vis spectrophotometer and a Hitachi F-4500 fluorescence spectrophotometer, respectively. DSC measurement was studied on a TAQ20 instrument operated at a heating rate of 10 °C/min from 25 °C to 300 °C in N_2. TGA was recorded on a Thermogravimetric Analyzer at the heating rate of 10 °C/min from 30 °C to 700 °C in N_2. The transient PL spectrum was performed by FLS980 fluorescence spectrometer. X-ray diffraction spectra were recorded on a Rigaku Miniflex 600 with 2θ range of 10°-75°, scanning at a rate of 1°/min.

4. PL in Different Solvents

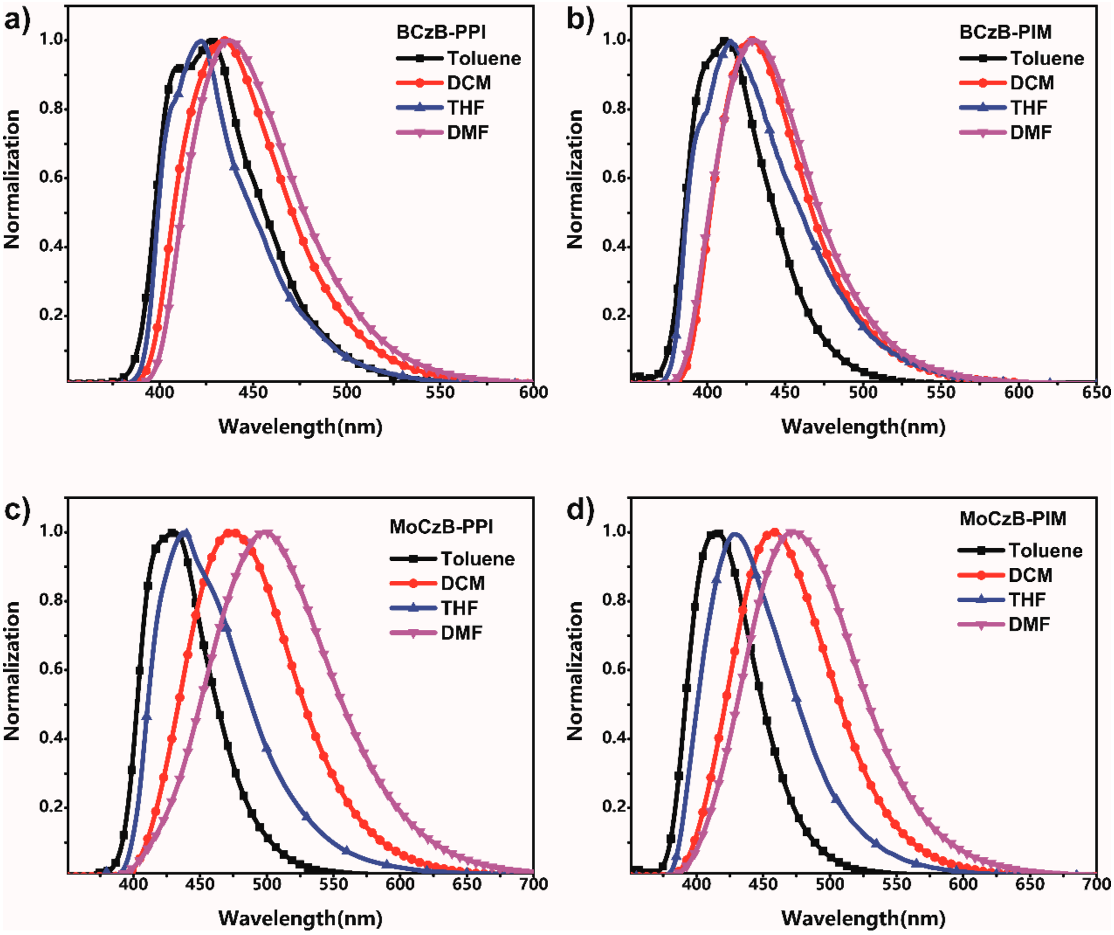

Figure S1. Normalized emission of BCzB-PPI(a), BCzB-PIM(b), MoCzB-PPI(c) and MoCzB-PIM(d) in different solutions: toluene, DCM, THF, and DMF.

5. PL Decay Behavior

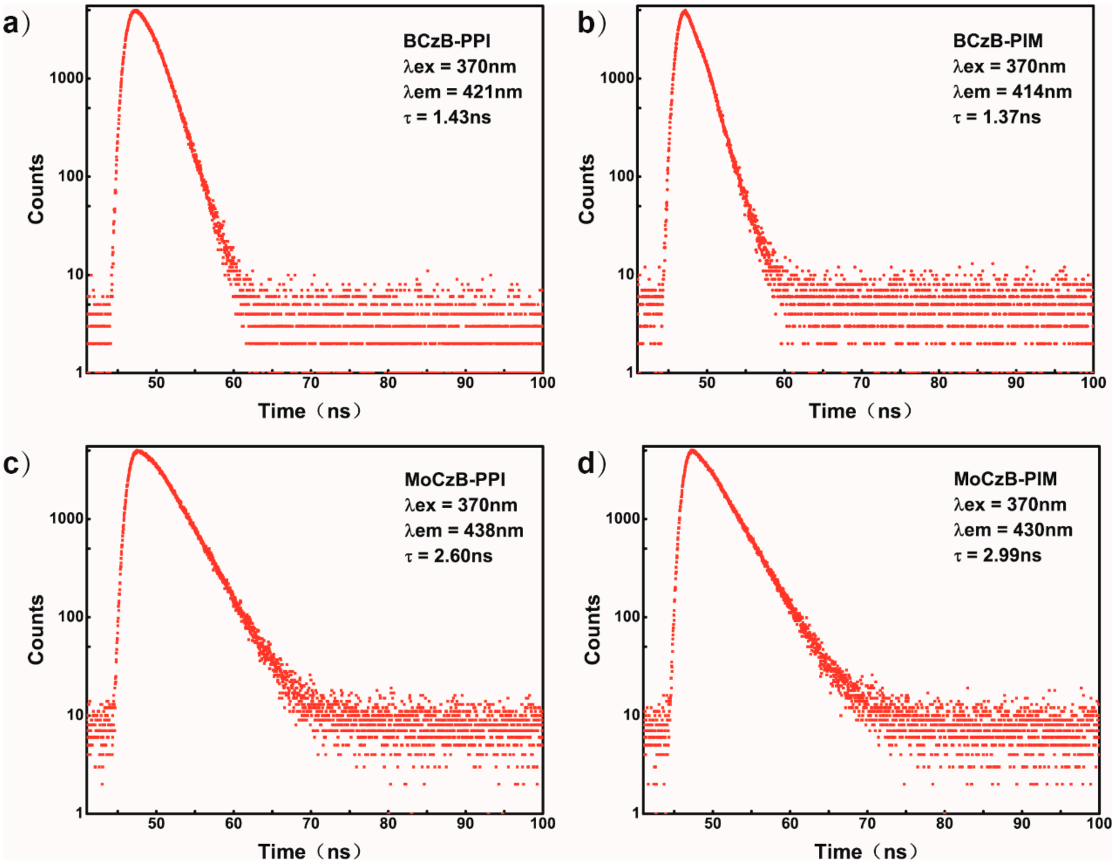

Figure S2. The decay behavior of BCzB-PPI (a), BCzB-PIM (b), MoCzB-PPI (c) and MoCzB-PIM (d) in degassed THF by using time-correlated single photon counting method.

6. Electrochemical Properties

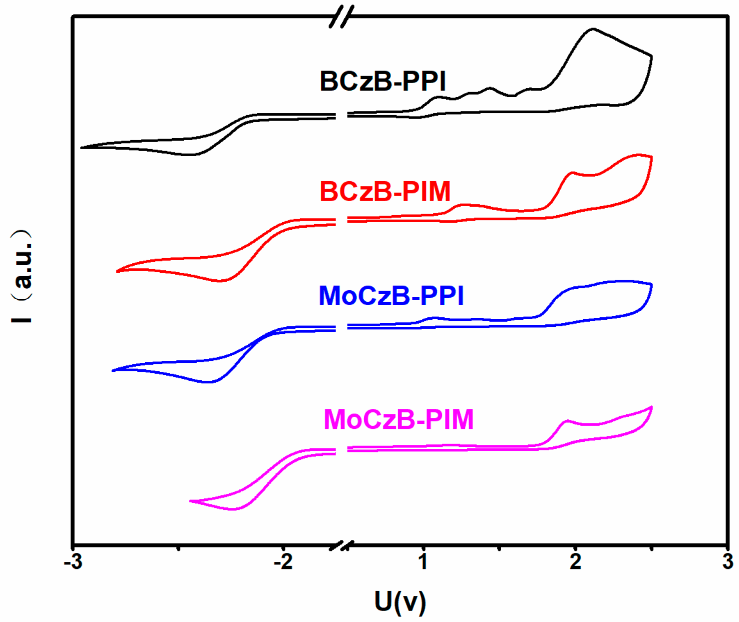

Figure S3. Cyclic voltammograms of four compounds, the potentials are calibrated against Fc/ Fc+ internal standard.

Cyclic voltammetry measurements were conducted in 1.0 mM concentrations of all molecules. Electrochemical studies were carried out in 0.1 M solutions of Bu4NBF4 in dichloromethane (DCM) solvent at room temperature. The electrochemical cell comprised of 3 mm diameter glassy carbon disk as a working electrode, an Ag/AgCl electrode as a reference electrode and a platinum wire as an auxiliary electrode. Cyclic voltammetry measurements were conducted at room temperature at a potential rate of 50 mV/s. We used ferrocene/ ferrocenium (Fc/Fc+) redox couples as internal standard. Finally, all the potentials relative to Ag/Ag electrode measured by CV were referenced against Fc/Fc+ to calculate the HOMO/LUMO energy levels. Thereby eliminating the uncertainty of Ag/Ag electrode. The HOMO/LUMO energy levels were calculated according to the following formalism: $E_{HOMO} = -(E_{OX}\ vs.F_c/F_c^+ + 4.8)eV$; $E_{LUMO} = -(E_{red}\ vs.F_c/F_c^+ + 4.8)eV$ where the Eox vs. Fc/Fc+ and Ered vs. Fc/Fc+ are oxidation and reduction onset potentials relative to Fc/Fc+ internal reference, respectively.

7. Device Fabrication and Measurement

ITO-coated glasses with a sheet resistance of 30 Ω per square were used as the substrates. Before manufacturing the device, the ITO glass substrate was cleaned with detergent, deionized water, ethanol, acetone and isopropanol in sequence, and finally cleaned with ethanol and boiled for 5 minutes, then dried with argon and treated with oxygen plasma for 10 minutes. The PEDOT:PSS layer were spin-coated and annealed at 140°C for 15 minutes. Then, NPB, EML and TPBi organic functional layers are sequentially vacuum deposited. Finally, 1nm LiF and 100nm Al were deposited. A Konicaminolta CS-2000 was used to measure the EL spectrum, device brightness and CIE coordinates. Keithley 2400 SourceMeter were used to record current-voltage characteristics under ambient atmosphere. Due to the sensitivity of OLED devices to water and oxygen, all tests on OLED devices were performed in glovebox under N2.

8. Performance of Device

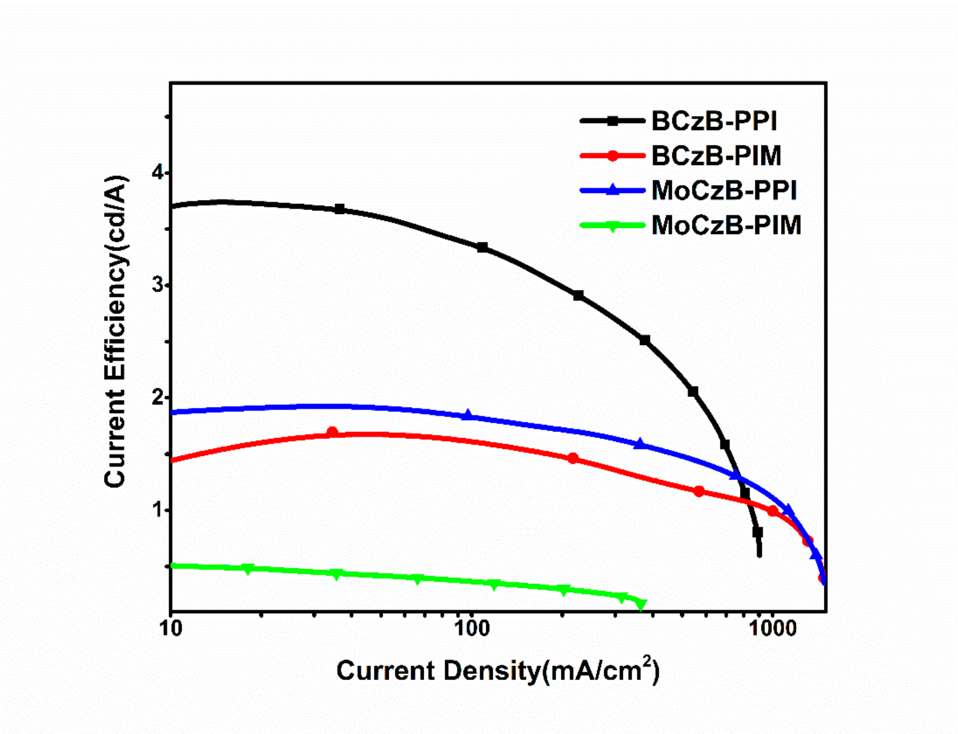

Figure S4. Current density-current efficiency curves.

9. Lifetime of Device

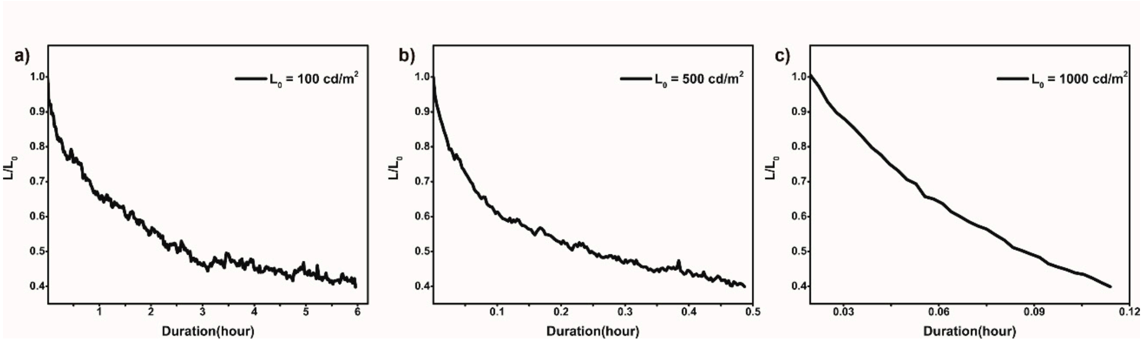

Figure S5. The lifetime (T_{50}) of BCzB-PPI OLED at initial luminance of 100(**a**), 500(**b**) and 1000 cd/m^2(**c**).

10. NMR

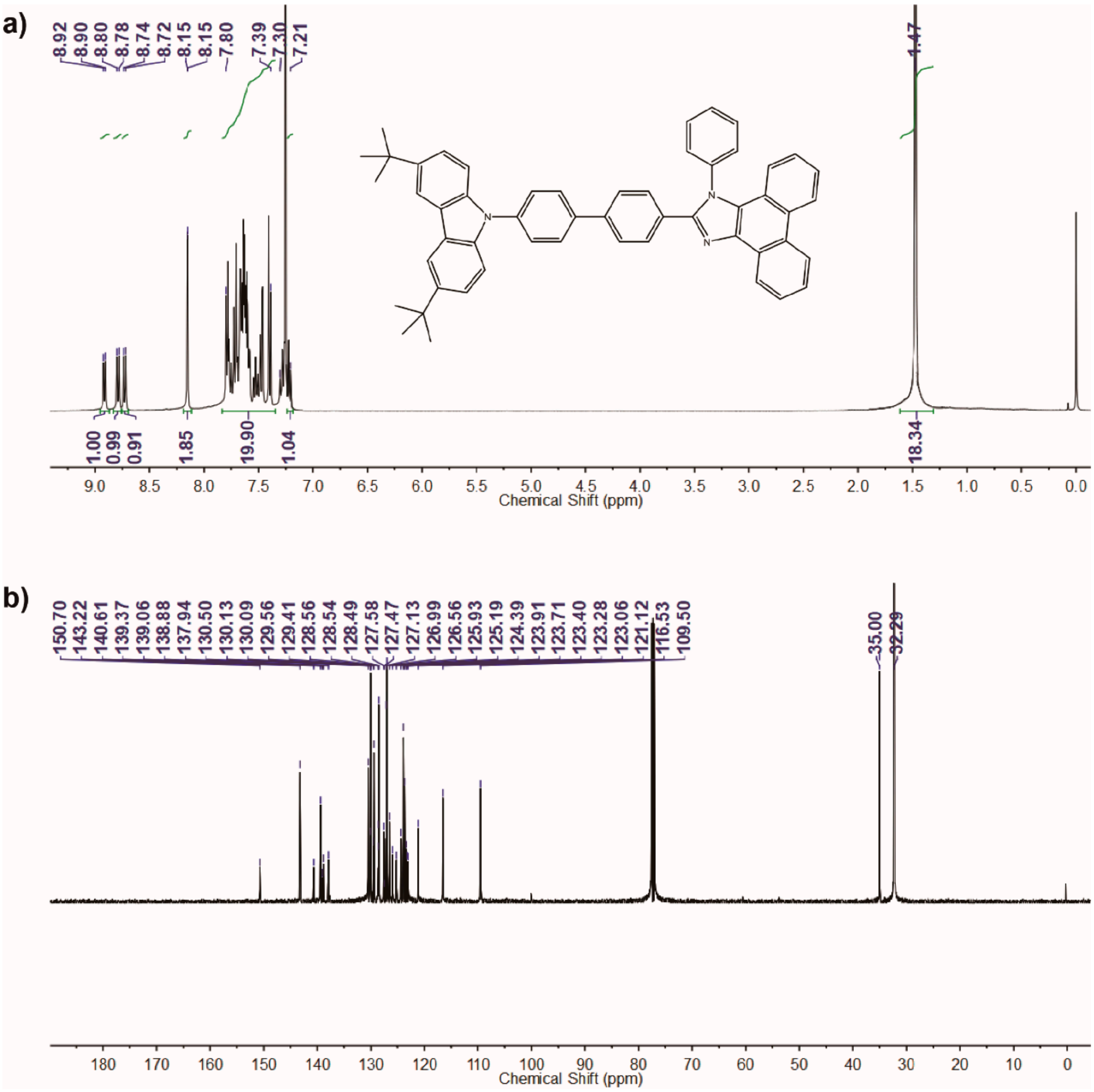

Figure S6. ^{1}H NMR(**a**) and ^{13}C NMR(**b**) of BCzB-PPI.

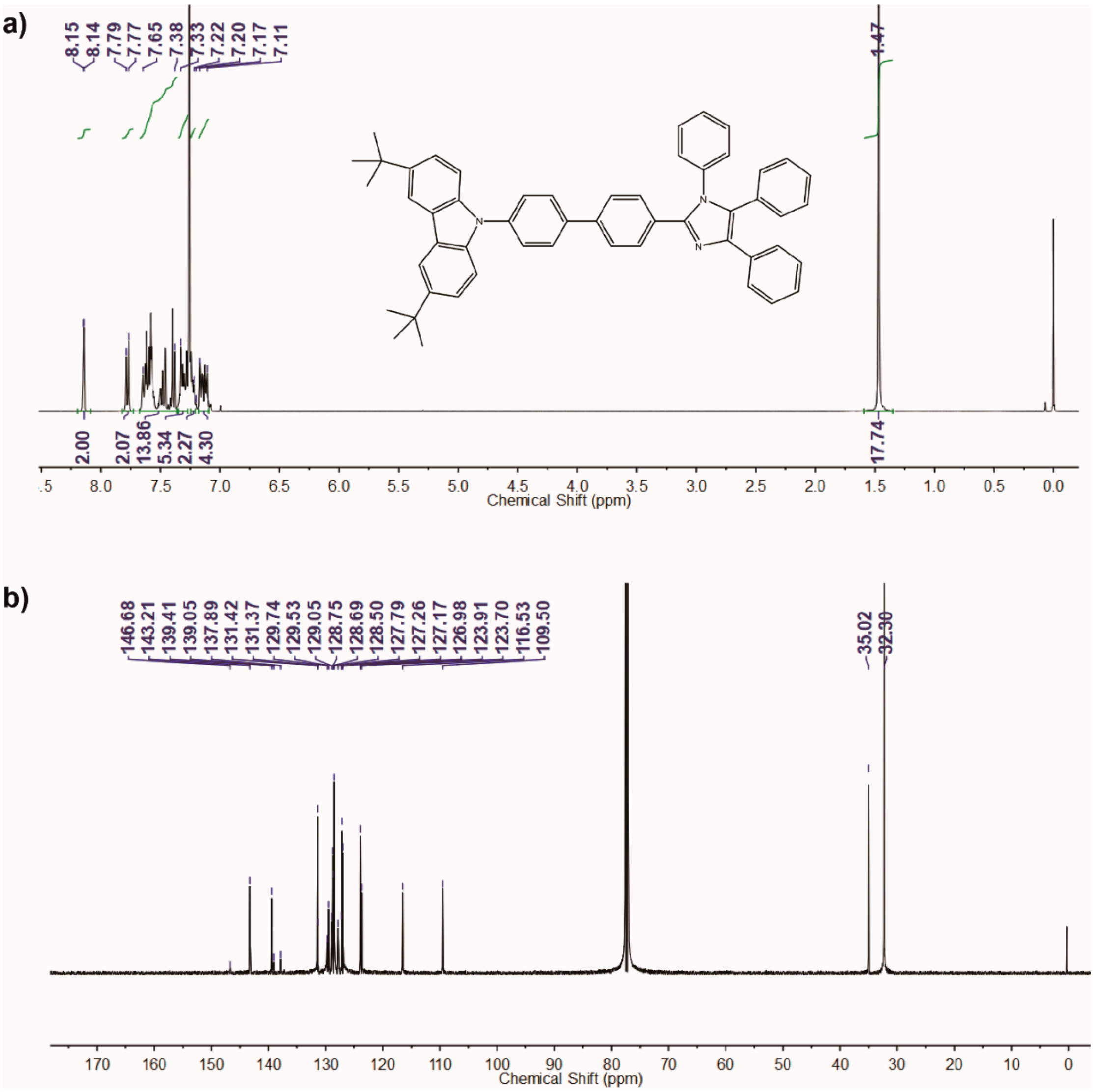

Figure S7. ^{1}H NMR(**a**) and ^{13}C NMR(**b**) of BCzB-PIM.

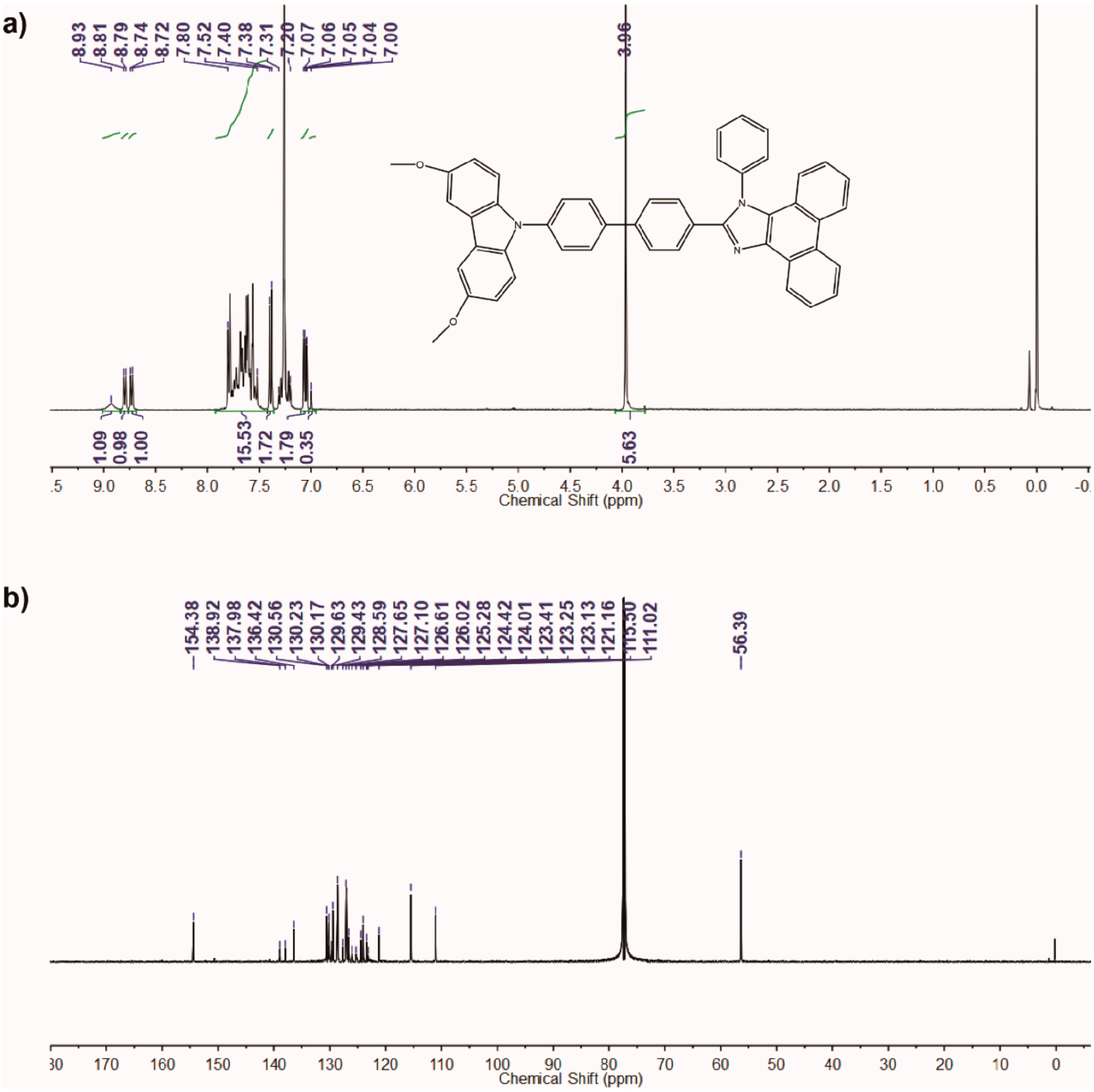

Figure S8. ^{1}H NMR(**a**) and ^{13}C NMR(**b**) of MoCzB-PPI.

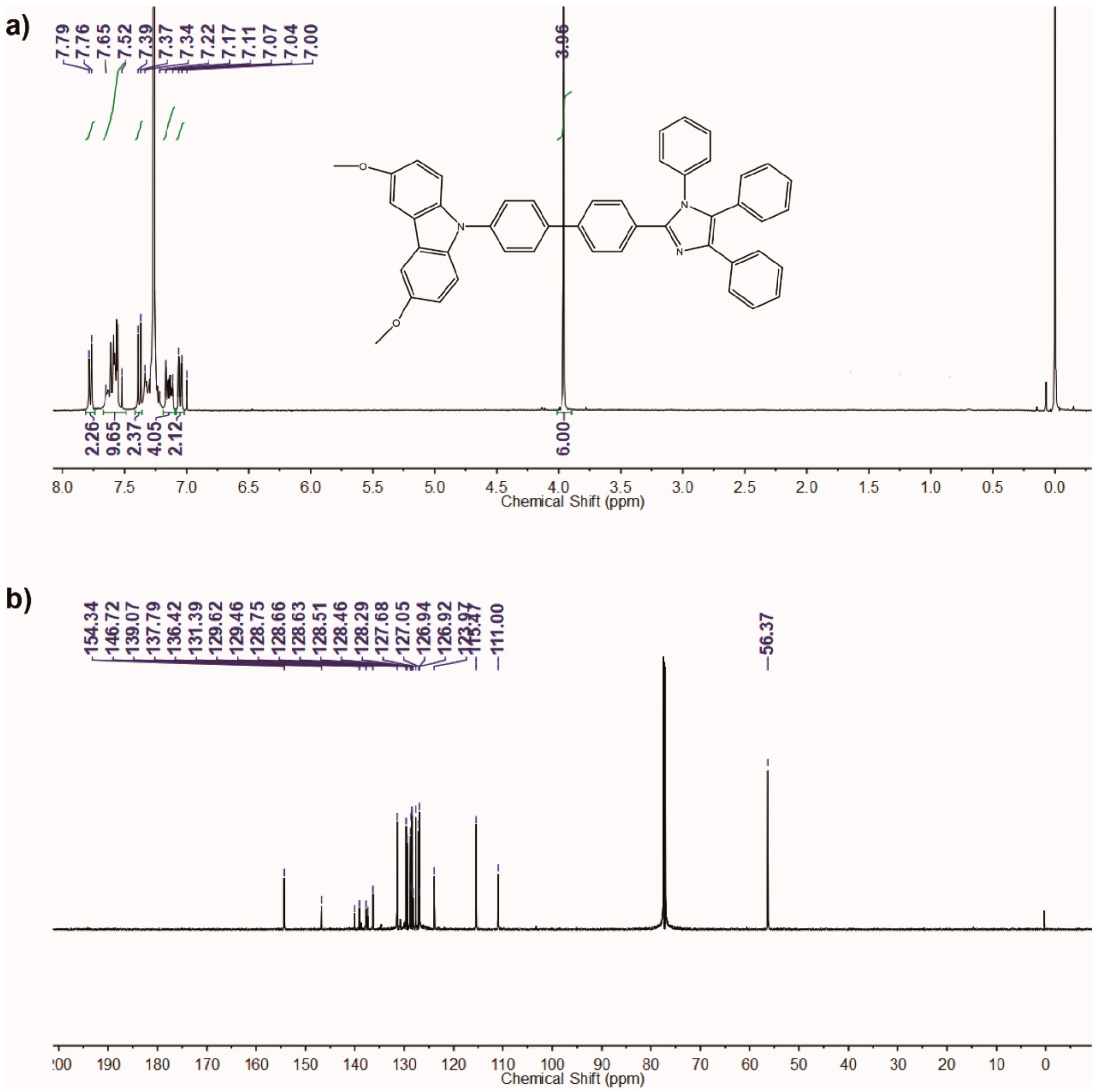

Figure S9. ^{1}H NMR(**a**) and ^{13}C NMR(**b**) of MoCzB-PIM.
